# Hemp Fiber Reinforced Red Mud/Fly Ash Geopolymer Composite Materials: Effect of Fiber Content on Mechanical Strength

**DOI:** 10.3390/ma14030511

**Published:** 2021-01-21

**Authors:** Eyerusalem A. Taye, Judith A. Roether, Dirk W. Schubert, Daniel T. Redda, Aldo R. Boccaccini

**Affiliations:** 1 Department of Materials Science and Engineering, Institute of Biomaterials, University of Erlangen-Nuremberg, Cauerstrasse 6, 91058 Erlangen, Germany; eyerusalem.taye@fau.de; 2 Institute of Technology, School of Mechanical and Industrial Engineering, Addis Ababa University, Addis Ababa 7754, Ethiopia; daniel.tilahun@aait.edu.et; 3 Department of Materials Science and Engineering, Institute of Polymer Materials, University of Erlangen-Nuremberg, Martensstr 7, 91058 Erlangen, Germany; judith.roether@fau.de (J.A.R.); dirk.schubert@fau.de (D.W.S.); 4 Bavarian Polymer Institute, Key Lab Advanced Fiber Technology, 90762 Fürth, Germany

**Keywords:** hemp fiber, geopolymer composite, mechanical properties, red mud, fly ash, microstructural analysis

## Abstract

Novel hemp fiber reinforced geopolymer composites were fabricated. The matrix was a new geopolymer based on a mixture of red mud and fly ash. Chopped, randomly oriented hemp fibers were used as reinforcement. The mechanical properties of the geopolymer composite, such as diametral tensile (DTS) (or Brazilian tensile) strength and compressive strength (CS), were measured. The geopolymer composites reinforced with 9 vol.% and 3 vol.% hemp fiber yielded average DTS values of 5.5 MPa and average CS values of 40 MPa. Scanning electron microscopy (SEM) studies were carried out to evaluate the microstructure and fracture surfaces of the composites. The results indicated that the addition of hemp fiber is a promising approach to improve the mechanical strength as well as to modify the failure mechanism of the geopolymer, which changed from brittle to “pseudo-ductile”.

## 1. Introduction

The sustainable development agenda has become a significant issue worldwide within the public as well as in the industrial sectors [1]. The need for considering environmental aspects when designing and developing new materials plays an increasing and greater role, in particular, in order to include the vital issues of limiting environmental pollution and preserving natural resources [2,3,4]. Extensive amounts of waste materials and by-products are generated by industrial processes, by the commercial sector, and by the general public every year, which are becoming increasingly difficult to handle with currently available technologies, thus, novel solutions are urgently required to achieve sustainable economic development [5]. Currently, researchers are working on the utilization of industrial wastes and by-products ranging from agricultural to plastic waste in a range of value-added applications [6,7,8].

Geopolymerization is a material processing technology that can transform, using low-temperature processes, waste materials that contain silica and alumina into useful products with excellent physical and chemical properties [8,9]. In recent years, there has been increasing interest in the utilization of industrial by-products such as fly ash [10], blast furnace slag [11], rice husk ash [12], and red mud [13] for the synthesis of geopolymer materials.

Geopolymers are eco-friendly and sustainable alternatives to conventional cement-based construction materials [14]. Owing to their attractive properties, geopolymers are being considered for a wide variety of applications, for example, as refractories, adhesives or structural parts [15,16], protective coatings [17], thermal insulating foams [18], high-tech ceramics [19], fire-proof building materials [20], and hybrid inorganic composites [21]. Moreover, due to their economic production process, many studies have been carried out on the use of geopolymers for mechanical components in industry [22]. However, geopolymers exhibit brittle behavior with relatively low tensile strength and are sensitive to microcracking. These drawbacks limit their use in load-bearing applications and affect the long-term durability behavior of geopolymer parts. Researchers have proposed the addition of natural fibers to geopolymer matrices to improve mechanical behavior, in particular, brittleness and tensile strength [23,24,25,26]. In the current study, fly ash, red mud, and hemp fibers were used to produce novel geopolymer composites. Fly ash is an industrial silicate waste produced by coal-fired power plants and collected during flue gas cleaning. It is one of the numerous inorganic substances that require specialized treatment before its safe disposal to avoid air and soil pollution [27]. Red mud, on the other hand, is a solid toxic waste that is generated during the Bayer process for the extraction of alumina from bauxite [28]. There are an estimated three billion tons of red mud accumulated globally, and the amount is increasing by approximately 120 million tons each year [29]. These huge amounts of red mud may cause groundwater contamination through percolation; this waste material is categorized as “hazardous” [30], thus requiring safe disposal. Hemp fibers are eco-friendly, exhibit relatively high specific strength, and are lower in cost, compared to synthetic fibers [31]. The aim of this study was to evaluate the performance of a new family of red mud/fly ash based geopolymer composites reinforced with different volume percentages of hemp fiber. Recently, hemp fiber has been receiving widespread attention since it shows rapid growth, a large degree of dry biomass production, and a high carbon storage potential [32].

To date, very few studies have investigated the usage of hemp fibers to reinforce fly ash based geopolymer matrices. Korniejenko et al. [33] and Maichin et al. [34] used sodium hydroxide treated hemp fibers to reinforce fly ash based geopolymers. The researchers observed that the hemp fibers were well suited for the fabrication of geopolymer composites and led to an improvement of mechanical properties compared to the unfilled geopolymer.

Moreover, the presence of the hemp fibers changed the character of the failure mechanism from brittle to quasi-ductile. Likewise, Sikapizye and Habanyama [35] used hemp fibers in fabric form in their study to produce fly ash based geopolymer composites and found that the highest flexural strength of the composites was 7.9 ± 0.2 MPa. In the present study, the fly ash used for the production of geopolymer composites was partially substituted with 30 wt.% of red mud to study the possibility of making a novel geopolymer composite incorporating a combination of challenging waste materials. To the best knowledge of the authors, no study has previously been published on hemp fiber reinforced red mud/fly ash geopolymer composites.

## 2. Materials and Methods

A geopolymer matrix was prepared by combination of 70% low calcium fly ash (Class F according to ASTM C618) and 30% red mud. The mean particle size of fly ash was d- (0.5) = 21μm. The red mud had a particle size less than 75 µm. Commercial sodium silicate solution containing 32% SiO_2_, 16% Na_2_O, and 52% water by mass (supplied by PQ Corporation, Eijsden, The Netherland) was used as the alkaline solution, and sodium hydroxide pellets with 99% purity (Roth GmbH, Karlsruhe, Germany) were procured and used without further purification. The hemp fiber was provided by BAFA Neu GmbH, Malsch, Germany. The fibers were washed in hot water (100 °C) and dried in an oven at 50 °C for 24 h prior to usage. Table 1 summarizes the chemical compositions of the fly ash and red mud used in the present experiments.

### 2.1. Sample Preparation

The alkali activator was prepared by mixing Na_2_SiO_3_ solution and 8M NaOH solution (in a ratio of 2.5) for 24 h prior to use. The alkaline liquid solution to solid ratio was 0.5 by mass. The geopolymer matrix was prepared by mixing fly ash, red mud, and the alkali solution using a laboratory motorized mixer at 700 rpm for 30 min. Subsequently, chopped hemp fibers with lengths of 10–13 mm were gradually added and mixed until a homogeneous slurry was obtained. Fiber contents of the four composites investigated in this study were 3, 6, 9, and 12% (by volume of solid material); unreinforced geopolymer (0% fiber content) was prepared as a control.

### 2.2. Compressive Strength

**Figure 1:**
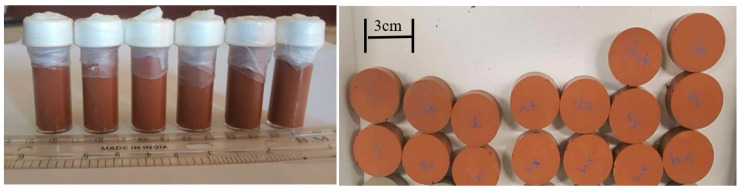
Geopolymer composite samples prepared for compressive (**left**) and Brazilian (diametral tensile) strength (**right**) tests.

The compressive strength test was performed on cylindrical samples with an aspect ratio (i.e., height to diameter) of 2:1 (diameter =14 mm, height = 28 mm) (Figure 1, left). The compressive strength test was carried out according to ASTM C39 (standard test method for compressive strength of cylindrical concrete specimens). A minimum of five samples were tested for each batch. The samples were tested to evaluate the 28 days compressive strength of the geopolymer composite. A universal testing machine (Instron 2530-30KN/139912 5967M124) was used applying a load at a cross-head speed of 0.5 mm/min with a load cell of 30 kN until the specimen failed. The compressive strength (σ_c_) of a sample was calculated using the following formula:

(1) $$\sigma_{c}=\frac{F}{A}$$

where F is the force on the sample at the point of failure and A is the area of the bearing surface of the specimen.

### 2.3. Scanning Electron Microscopy (SEM)

The adhesion between the fibers and the geopolymer matrix was analyzed qualitatively via scanning electron microscopy (LEO 435 VP; LEO Microscopy Ltd., Cambridge, UK, and Ultra Plus; Zeiss, Jena, Germany). Fracture surfaces were also observed by SEM.

### 2.4. Brazilian Tensile Strength Test

The Brazilian indirect tensile strength (or diametral tensile strength) was measured by using an Instron 2530-30KN universal testing machine (detailed above). The test was carried out according to the methodology described in the ASTM D3967-16 (tensile strength of disk-shaped specimens). The diameter-to-thickness ratio was two (diameter = 30 mm, thickness = 15 mm), and five samples for each batch mixture were tested (Figure 1, right). The Brazilian tensile strength of the sample was calculated using the following formula:

(2) $$\sigma_{t}=\frac{2F}{\pi \mathrm{Dt}}$$

where σ_t_ is the Brazilian tensile strength (MPa); F is the maximum force on the specimen (N), D is the diameter (mm); and t is the thickness of the specimen, measured at the center (mm). The test was carried out at room temperature; the cross-head speed applied was 0.5 mm/min.

## 3. Results and Discussion

### 3.1. Compressive Strength

**Figure 2:**
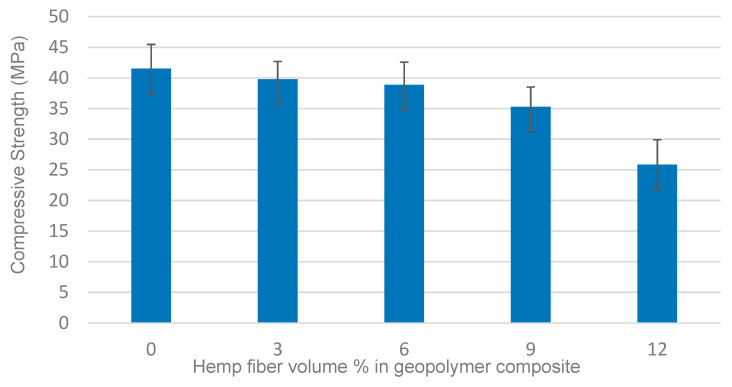
Effect of hemp fiber content on compressive strength of geopolymer composites.

Figure 2 shows the effect of increasing hemp fiber content on the compressive strength of the composites. Samples reinforced with hemp fibers exhibited a low reduction of compressive strength for fiber vol. fractions < 6%, which might be explained by the low cohesiveness between the fibers and the geopolymer matrix. In comparison with the unreinforced material, the addition of 3 vol.% fibers showed only a marginal (non-significant) reduction of the compressive strength. However, the compressive strength of the composite was reduced to a larger extent when the fiber content was increased, possibly because higher contents of hemp fibers (>9 vol.%) led to reduced homogeneity of the distribution of fibers within the matrix and possibly to fiber–fiber contact, which is detrimental to the mechanical properties. Indeed, fiber agglomerations may act as stress concentration points, thus reducing the stress required to fracture the specimen. It was observed that increasing the fiber content caused a reduction of workability of the matrix, which in turn allowed air bubbles to be introduced either through mixing or by being trapped in the geopolymer during pouring into the mold. On the other hand, the optimal loading of hemp fiber offers less potential for macro void formation, and a more uniform dispersion of fibers can be achieved, which contributes to the fracture strength improvement. The 40 MPa average compressive strength for red mud/fly ash geopolymer reinforced with 3 vol.% hemp fibers was comparable to the value found by Alomayri et al. for fly ash based geopolymer composites with 0.5 wt.% of cotton fiber [36] and it was higher than values reported by Ye et al. [37] for wood fiber reinforced fly ash containing geopolymer composite. However, the values found in this work are lower than those reported by Assaedi et al. [38] for 4.1 wt.% flax fabric reinforced geopolymers and by Korniejenko et al. [33] for 1 wt.% hemp fiber reinforced geopolymers.

### 3.2. Brazilian Tensile Strength

**Figure 3:**
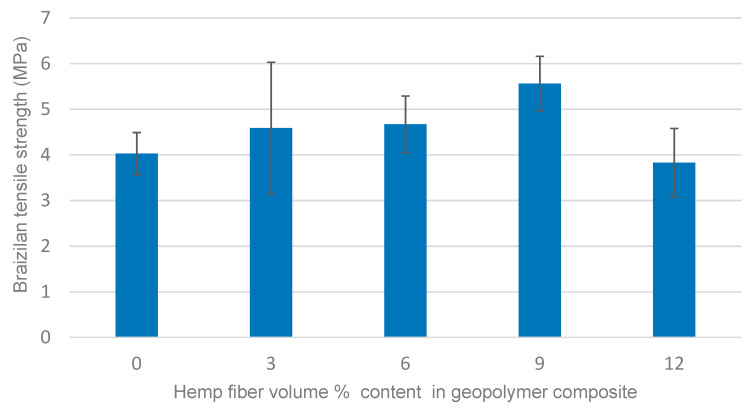
Effect of hemp fiber content on Brazilian tensile strength of geopolymer composites.

The effect of fiber content on the Brazilian tensile strength of the composites is shown in Figure 3. The experimental results indicate that the strength improved by increasing the content of hemp fiber. The value of the neat geopolymer was shown to improve by 28% with the addition of 9 vol.% of hemp fiber, but the addition of 12 vol.% of hemp fiber did not show any strength improvement, which might be attributed to the lack of the workability and cohesiveness between the fibers and the geopolymer matrix, as mentioned above. A similar trend has been observed in other studies. For example, Chen et al. [39] and Maichin et al. [34] in their studies confirmed that the addition of the optimum content of sweet sorghum and hemp fiber, respectively, led to a remarkable improvement in the tensile strength of the composites.

### 3.3. Load versus Displacement Curves in Brazilian Tensile Strength Test

**Figure 4:**
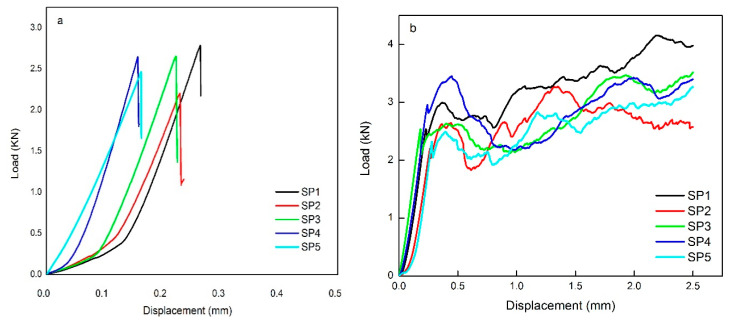
Typical load versus displacement curves obtained by Brazilian tensile strength test of (**a**) unreinforced geopolymer and (**b**) geopolymer composites with 9 vol.% hemp fibers.

Figure 4a,b shows the load versus displacement curves corresponding to the Brazilian tensile strength test of neat geopolymer and geopolymer composite samples reinforced with 9 vol.% hemp fibers, respectively. The load-displacement curves show notable differences.

Figure 4a shows the load versus displacement curves of the plain geopolymer material displaying a linear behavior and sudden fracture at the peak load. On the contrary, Figure 4b shows that the incorporation of hemp fiber (9 vol.%) leads to a non-linear behavior, and the maximum peak load increases.

It was observed that none of the fiber-reinforced specimens were entirely broken at the peak load. This could be attributed to the crack bridging effect of the hemp fibers under load, which results in an increase of the tensile strength in comparison to that of the unreinforced geopolymer samples. Fibers are able to withstand a higher load and are capable of supporting the development of multiple cracks through the loading process, consequently preventing the brittle failure of the matrix. The failure mechanism was changed from brittle to “pseudo-ductile.”

### 3.4. Scanning Electron Microscopy

**Figure 5:**
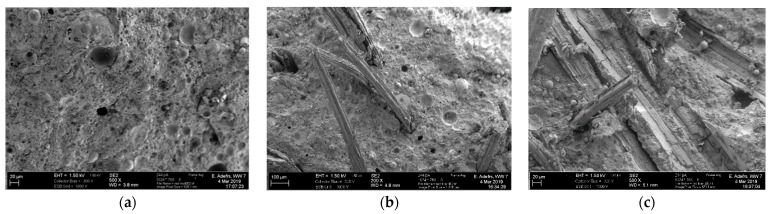
SEM images (fracture surface) of (**a**) plain geopolymer, and (**b**,**c**) geopolymer reinforced with hemp fiber (9 vol.%) and prepared by 8M NaOH at two different magnifications.

Figure 5 shows representative SEM images of the fracture surface of a 9 vol.% hemp fiber reinforced geopolymer specimen after the Brazilian tensile strength test. The main toughening mechanism appears to be fiber pull-out, as indicated by the fiber topography on the fracture surfaces. Moreover, the SEM images indicate qualitatively that there was an effective wetting between the hemp fibers and the red mud/fly ash geopolymer matrix, as evidenced by the imprints of the fiber morphology on the matrix surface. This in turn means that friction would occur between the fiber and the matrix during fracture, which is an energy dissipating mechanism contributing to fracture toughness increment.

## 4. Conclusions

This study showed that hemp fibers are suitable for the reinforcement of red mud/fly ash based geopolymer composites. Mechanical characterization using the Brazilian (or diametral) tensile strength and compressive strength tests showed that the addition of hemp fiber improved the mechanical properties of the geopolymer composites. The increase in diametral tensile strength was particularly noticeable for composites reinforced with 9 vol.% of hemp fibers and synthesized with 8M of NaOH, which displayed a diametral tensile strength of around 5.5 MPa. Additionally, SEM micrographs showed evidence of toughening mechanisms, i.e., fiber pull-out, as a factor contributing to the enhanced mechanical behavior of the hemp fiber reinforced geopolymer composite. With increasing fiber content, the compressive strength of composites was reduced, compared to unreinforced geopolymer, which was ascribed to the increasing inhomogeneous distribution of fibers in the matrix. The use of hemp fibers appears as an effective strategy to strengthen and toughen geopolymer matrices based on industrial wastes, leading to eco-friendly alternative building materials. However, durability, cytotoxicity, and leaching studies are required to assess the safety of the new composites for construction applications.

## Figures and Tables

**Table 1:** Chemical composition of the raw materials used in this study.

Chemical Composition (in wt %)	SiO_2_	Al_2_O_3_	Fe_2_O_3_	CaO	Na_2_O	K_2_O	TiO_2_	Loss of Ignition
Fly ash (%)	54.36	24.84	8.28	2.56	0.83	3.03	1.07	2.04
Red mud (%)	5.21	15.21	52.94	2.65	2.40	0.63	8.05	10.77

## Data Availability

The data presented in this study are available on request from the corresponding author.
